# Characteristics of the different HIV-1 risk populations based on the genetic transmission network of the newly diagnosed HIV cases in Jiangsu, Eastern China

**DOI:** 10.1016/j.heliyon.2023.e22927

**Published:** 2023-11-28

**Authors:** Ying Zhou, Jing Lu, Zhi Zhang, Qi Sun, Xiaoqin Xu, Haiyang Hu

**Affiliations:** Institute of AIDS/STD Control and Prevention, Jiangsu Provincial Center for Disease Control and Prevention, Nanjing, 210009, China

**Keywords:** HIV-1, Genetic transmission network, Newly-diagnosed, Risk population

## Abstract

**Introduction:**

The HIV-1 prevalence has been steadily increasing in Jiangsu, China. HIV-1 genetic transmission network can be used to explore the transmission kinetics and precision intervention in high-risk populations. Thus, we generated an HIV-1 genetic transmission network, explored key risk populations based on different risk factors and found out the risk factors for HIV-1 prevention and control among the newly-diagnosed HIV-1 cases from 2017 to 2018.

**Method:**

We amplified the HIV-1 *pol* sequences from the plasma samples of the newly-diagnosed HIV-1 cases from 2017 to 2018 and obtained the infection data from The National HIV/AIDS Surveillance System. HIV-Trace and Cytoscape Software were both used to construct the HIV-1 genetic network with a gene distance of <0.005. The R software was used to analyze the risk factors for inclusion into the network.

**Results:**

We obtained 3362 sequences with the *pol* gene region, of which 3316 contained detailed individual information. CRF01_AE accounted for 42.3 % of the HIV-1 subtypes in the samples. The median CD4^+^T lymphocyte count was 329 cells/μL in 2017 and 313 cells/μL in 2018. At the gene distance threshold of 0.005, 481 sequences were incorporated into the HIV-1 gene network, constructing 202 clusters. Age over 60 years old, heterosexual transmission route, subtype (CRF105_0107, CRF55_01 B, and CRF67_01 B) and CD4^+^T lymphocyte count (>200) were the risk factors influencing inclusion into the HIV-1 gene network. Moreover, south Jiangsu cities had higher inclusion in the network. Thus, key risk populations in the clusters with different transmission routes, new emerging subtypes, drug resistance nodes, and individuals above 60 years of age in the network represented the critical risk populations that should be focused more on for intervention.

**Conclusion:**

The HIV-1 genetic transmission network is adept at discovering undiagnosed HIV-infected cases and linking all diagnosed cases for determination of risk infections. Therefore we should pay more attention to these risk infections with further investigation and intervention, helping to achieve the goal of 95 % use combination prevention from the World Health Organization, and push to end AIDS epidemic.

## 1 Introduction

HIV-1 is still a public health concern worldwide, even though antiretroviral therapy (ART) can inhibit viral replication and prolong life expectancy. The HIV epidemic has been gradually increasing in China, as shown by the number of people (1.14 million) living with HIV and new HIV diagnoses (approximately 133,000) reported in 2021 [1,2]. Jiangsu, an economically developed province in China, had a steady increase in new HIV-1 diagnoses in the last three years, with about 4000 infections annually [3]. However, the actual number of new diagnoses is underestimated, considering the effect of the COVID-19 epidemic. Many prevention strategies have been proved to be effective in controlling the spread of HIV among different high-risk populations. Unfortunately, implementing these interventions to all high-risk populations is often infeasible in various settings [4]. Thus, it is imperative to find out key risk populations from a new perspective for further a new precision intervention [5,6]. An increasing number of studies support that molecular network analyses could contribute to reveal among whom and to where the HIV infection is spreading and to estimate the speed of HIV transmission. It was a break to be widely used in recent years in HIV-1 transmission kinetics and high-risk populations' precision intervention research. Several aspects have been incorporated into the network to illustrate characteristics of the HIV-1 spread at the population level, thus providing critical information to help improve the case-finding strategies and activity control of critical groups in the network [[7], [8], [9]].

Studies on HIV prevalence in Jiangsu have focused more on the male-to-male sexual contact (MMSC) group, which only accounts for half of the HIV infection cases [10,11]. To conduct detailed individual analysis of the new HIV-1 diagnoses and identify more undiagnosed infections and more HIV-negative network members at high risk of infection, we used this molecular network-based strategy. In spite of MMSC group, we also pay attention to the role played by females or people over 60 years old in the genetic transmission network. All these conductions are to achieve the first 95 % target of UNAIDS that could be a part of the global push to end the AIDS epidemic by 2030.

## 2 Materials and methods

### 2.1 Laboratory detection

The newly-diagnosed HIV-1 whole blood samples between 2017 and 2018 were collected for sequences amplification. The sequences further were used to construct the Jiangsu HIV genetic transmission network. The CD4^+^T lymphocyte counts (CD4 counts) were completed within 24 h after the sample collection. The whole blood samples were centrifuged at 3000 r/min for 15 min to collect plasma for HIV-1 gene test. Genotypic drug resistance (DR) was detected via RT-PCR and Nest-PCR [12]. The PCR products were Sanger sequenced by the Sangon Biotech company (Shanghai, China). We obtained partial HIV-1 polymerase (*pol)* coding region, including the protease gene (1–99 amino acids) and the reverse transcriptase gene (1–270 amino acids). All sequences were submitted to the HIV DR database (https://hivdb.stanford.edu/) for the DR results including mutations and resistance levels to drugs. Sequences with low degree level or above resistance to any drugs against protease and reverse transcriptase regions were defined as DR sequences.

### 2.2 HIV genetic transmission network inference

The sequences were aligned on MEGA 7.0, with reference sequences downloaded from the HIV Sequence Database (https://www.hiv.lanl.gov/content/sequence/NEWALIGN/align.html). Aligned sequences eventually had 1000 bp bases. After the sequences were aligned with the reference sequences, the ML phylogenetic tree was constructed. The subtype of the sequences in the same branch with the reference strains was determined as the reference strain sequence subtype. The Hyphy software was used to compute the distance between the two sequences, and the sequences with a distance of <0.005 were used to generate an HIV-1 genetic transmission network via the HIV-1 Trace and Cytoscape Software following the protocol advised by China CDC.

### 2.3 Statistical analysis

The function “glm” of the R (version 4.1.3) package “rms” was used for statistical analysis. The univariate logistic analysis determined the associations between the variables and the formed network. The variables with p < 0.2 (from the univariate logistic analysis) were incorporated into the logistic regression model to determine the significance level via binary logistic regression. Variables with p < 0.05 were considered statistically significant.

### 2.4 Individual information

The individual demographic information were acquired from the National HIV/AIDS Surveillance System, including identification card (ID) number, gender, age, ethnicity, transmission route, education degree, marital status, occupation, the institutions where patients being discovered, the confirmation date of HIV-1 infection, CD4^+^T cell counts before on ART.

## 3 Results

### 3.1 Information of the study population

**Fig. 1:**
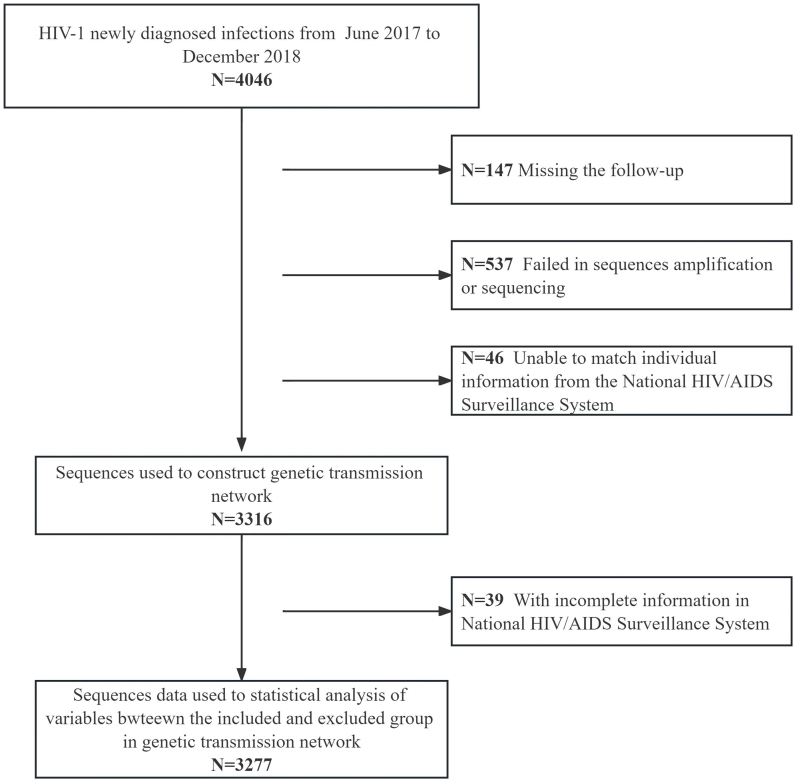
The flow chart of individuals included in the study.

We obtained 3362 sequences, among which 3316 had detailed individual information accessed from the National HIV/AIDS Surveillance System (Fig. 1). Moreover, 87.7 % of the infections were male, with a median age of 36 (interquartile range [IQR], 27–50). Nearly half of the individuals were married, 22.2 % of the population worked as commercial service providers, 19.2 % were farmers, and 17.2 % were housekeeping workers. The population percentage with education level of high school or above was 54.4 %. Furthermore, the transmission route was mostly through sexual transmission, with MMSC accounting for 60.3 %. The median CD4 count was 320 cells/μL (IQR: 204–464). Patients diagnosed at the voluntary counseling and testing (VCT) centers and the comprehensive hospitals accounted for 42.9 % and 38.0 %, respectively. The main circulating subtypes were CRF01_AE and CRF07_BC, accounting for 42.6 % and 31.1 %, respectively. Moreover, 90 % of the HIV-1 sequences were sensitive strains without any drug resistance mutation.

### 3.2 HIV genetic transmission network

HIV-1 *pol* sequences from 3316 HIV-infected individuals were used to infer an HIV genetic transmission network. Eventually, the network consisted of 399 putative transmission links involving 15 % (481/3316) of individuals overall and 6 % (196/3316) and 9 % (285/3316) of individuals in 2017 and 2018, respectively. There were 202 connected clusters in the network, among which 39 had at least 3 nodes, denoted as big clusters. The largest cluster had 16 nodes, and the following clusters had 9, 7, and 6 nodes. In addition to the big clusters described above, there were 2 clusters each with 5 nodes, 8 clusters each with 4 nodes, and 24 clusters each with 3 nodes.

### 3.3 Characteristics of the key population in the unique HIV-1 clusters of transmission network

**Fig. 2:**
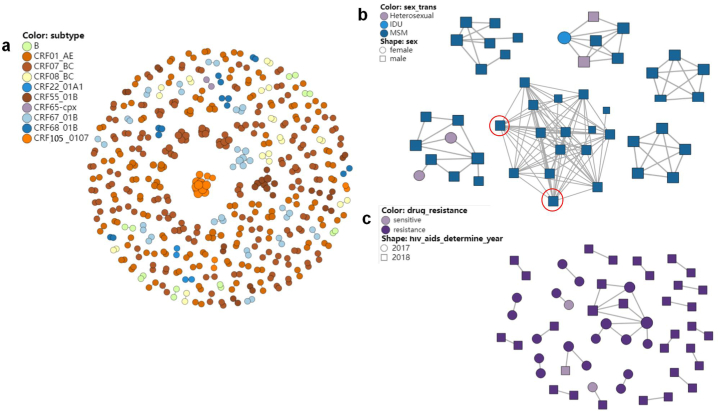
The HIV-1 genetic transmission network. 2a: The subtype clusters exhibition in HIV-1 genetic transmission network. 2 b: The exhibition of top six clusters in number of nodes through transmission route. The nodes with the most of 14° were red circled. 2c: The exhibition of clusters consisted of resistance mutations.

We first present a complete genetic network of the sequences subtype from 2017 to 2018 (Fig. 2a). In this genetic network, the sequences of subtype CRF105_0107 constructed three clusters, one of which was the largest cluster in the network with 16 nodes. The subtype of the second largest cluster with 9 nodes in the network was CRF55_01 B, and the sequences of subtype CRF55_01 B totally constructed 10 clusters. The subtype of the third largest cluster with 7 nodes was CRF67_01 B, and the sequences of subtype CRF67_01 B constructed 15 clusters in this network. HIV-1 subtypes in these three big clusters were all emerging subtypes in Jiangsu. The subtype with the most numerous clusters in the network was CRF07_BC (75 clusters), followed by CRF01_AE (72 clusters). The transmission route presented in the genetic network is an important way to identify individuals at high risk of HIV transmission. In this study, We highlighted the clusters with numbers of nodes in top 6 in the network (Fig. 2b). Among these 6 clusters, 4 clusters exhibited the route of MMSC, and one of them is the largest cluster in the network, with two nodes each containing 14 links (marked in the red circles in Fig. 2b). The number of links was defined as nodal degree. The other two clusters contained heterosexual and injection drug user (IDU) transmissions. In the cluster with 9 nodes composed of heterosexual and homosexual transmission routes, one heterosexual female had one degree, and the other had two degrees. Moreover, in the cluster consisting of 6 nodes, composed of heterosexual, IDU and homosexual transmission routes, the two heterosexual males had six degrees (four for one and two for the other) and both connected to the IDU female. The IDU female had five degrees connected to the heterosexuals and MMSC males. Drug resistance is another element for assessing the cluster risk because of its declining effect on ART. In the network, there were 52 DR nodes. 8 sequences exhibited resistance to nucleotide reverse transcriptase (NRTI) and 45 sequences showed resistance to non-nucleotide reverse transcriptase (NNRTI). The mutations in NRTI region were M41L, S68G, A62V, D67 N and K219E, accounting for 3.85 % (2/52) each. The mutations in NNRTI region were much more. The most numerous mutation was V179E (27/52), followed by V106I (6/52), and the third was V179D (4/52). The other mutations were V179T (3/52), Y181C (3/52), K103 N (3/52), E138G (2/52), H221Y (2/52), respectively. Through the network, we found a cluster with all 9 CRF07_BC resistance nodes from the 2017 and 2018 samples, and the mutation in the cluster was V179E. Another cluster with 3 nodes contained two resistant nodes from the 2017 samples and one sensitive node from the 2018 samples, respectively. Additionally, the other 20 resistant transmission clusters occurred in pairs (Fig. 2c).

**Fig. 3:**
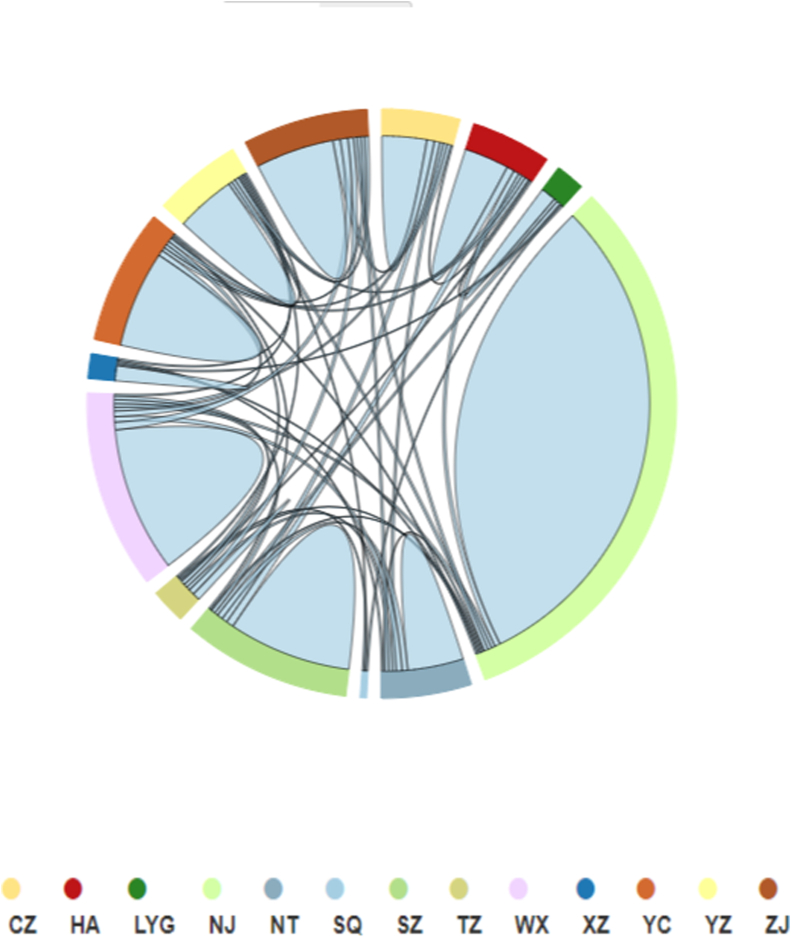
The distributions and associations of Jiangsu cities in the HIV-1 genetic transmission network.

The distributions of infections can also reflect the activity level in HIV-1 transmission. We showed the 13 cities in Jiangsu with their attributions in the network (Fig. 3). The connections between cities had a typical scheme. We found that the infections in cities mainly connected within cities. For example, 95 % (268/282) in Nanjing were mainly connected within Nanjing. 80 % (66/82) of those in Suzhou frequently interacted within Suzhou. Those infected with HIV in Nanjing, Suzhou and Nantong interacted with those in numerous cities because of the convenient transportation by the high-speed railway. Cities in north Jiangsu, like Xuzhou, Taizhou, Lianyungang, and Suqian, were highly associated with those in south Jiangsu. Thus, these results located cities with key risk populations.

**Fig. 4:**
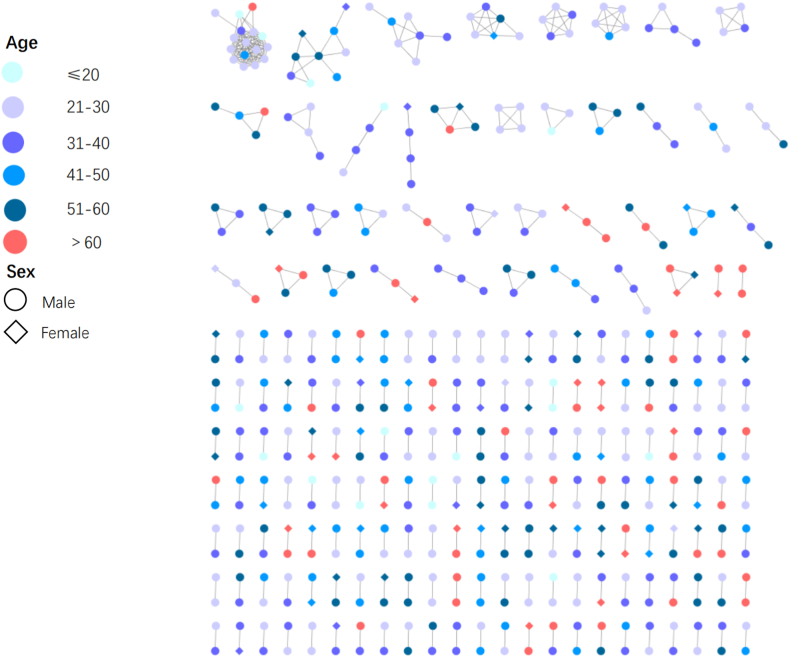
Clustering of different age groups in the HIV-1 genetic transmission network.

Older infections were becoming more and more in Jiangsu, and we constructed an age interval network using the Cytoscape software to visually present the transmission population based on age (Fig. 4). There were 59 nodes whose age was over 60 years in the genetic transmission network, among which 43 nodes were males, and 16 nodes were females. Of the 39 clusters with at least 3 nodes, only 10 clusters contained people over 60 years old, and the total number of people over 60 years old was only 15. The other nodes of individuals over 60 years old all showed in pairwise clusters. According to individual information, among 59 nodes, 47 nodes were involved in the heterosexual transmission, 11 were engaged in MMSC, and 1 was unknown but showed a pairwise connection with another MMSC. Moreover, in the transmission network, 21 males whose age was over 60 years connected with females, of which 14 were individuals over 60 years old. Two females had odd pairwise clusters in the network.

### 3.4 The analysis of sequences included in the genetic transmission network

To find the infection characteristics contributing to inclusion into the network, we compared the risk of variables included in the network with those not included (Table 1). We identified significant differences in age, transmission route, HIV-1 subtype, DR profiles, and CD4^+^ T cell count. Interestingly, individuals aged 21–40 and 41–60 were less likely to be incorporated into the network compared with individuals over 60 years old. Females were more likely to be included in the network, but had no significant difference. Unlike the sensitive viral hosts, resistant hosts had a higher risk of inclusion into the network. Compared with CRF01_AE, the CRF55_01 B and CRF67_01 B subtypes had a higher risk of evolving in the network. The pairwise comparison analysis showed that the difference in each CD4 count group was significant. We also found that a high CD4 count played a key role in inclusion in the network, as opposite to its protective role in HIV-1 infection immunity.

**Table 1:** The risk variables with statistical significance between the included and non-included groups in the HIV-1 transmission network.

Variables	Total number of cases (n = 3277)	Number of cases in the network	Rate of cases in the network%	One-factor analysis		Multi-factors logistic analysis	
				OR (95%CI)	*P* Value	OR (95%CI)	*P* Value
Gender							
Male	2876	409	14.2	1	Ref		Ref
Female	401	67	16.7	1.21 (0.912–1.605)	0.186*	1.279 (0.942–1.716)	0.108
**Age**					Ref		Ref
≤20	152	29	19.1	1.015 (0.617–1.670)	0.954	0.854 (0.503–1.428)	0.552
21–40	1736	249	14.3	0.721 (0.523–0.993)	0.045*	0.637 (0.457–0.900)	0.009^#^
41–60	1092	142	13.0	0.643 (0.458–0.904)	0.011*	0.594 (0.421–0.846)	0.003^#^
≥61	297	56	18.9	1		1	
**Ethnicity**							
Han	3209	470	14.6	1	Ref		
Others	68	6	8.8	0.564 (0.243–1.311)	0.183*		
**Educational degree**							
Junior high school and below	1491	205	13.7	1	Ref		
High school or technical secondary school	790	114	14.4	1.058 (0.826–1.355)	0.655		
college and above	996	157	15.8	1.174 (0.937–1.471)	0.163*		
**Marital status**							
Unmarried	1337	197	14.7	1	Ref		
Married with spouse	1474	215	14.6	0.988 (0.802–1.218)	0.912		
Divorced or widowed	466	64	13.7	0.921 (0.68–1.249)	0.597		
**Occupation**							
Clerk	232	41	17.7	1	Ref		
Commercial service	730	99	13.6	0.731 (0.491–1.089)	0.123*		
Worker	545	89	16.3	0.909 (0.605–1.366)	0.647		
Housekeeping and houseworker	562	69	12.3	0.652 (0.428–0.993)	0.046*		
Farmer	630	95	15.1	0.827 (0.554–1.236)	0.355		
Retired	113	17	15.0	0.825 (0.445–1.528)	0.541		
Student	172	33	19.2	1.106 (0.666–1.838)	0.697		
Others or unknown	293	33	11.3	0.591 (0.36–0.97)	0.037*		
**Transmission route**							
Homosexual transmission	1981	311	15.7	1	Ref	1	Ref
Heterosexual transmission	1263	164	13.0	0.801 (0.654–0.983)	0.033*	0.809 (0.654–0.997)	0.005^#^
IDU and others	33	1	3.0	0.168 (0.023–1.229)	0.079*	0.156 (0.009–0.740)	0.069
**Institutions where patients being discovered**							
Institution of blood donation and donor	111	15	13.5	1	Ref		
Dermatosis and STD clinics	386	62	16.1	1.225 (0.667–2.25)	0.514		
Medical examinations and others	127	15	11.8	0.857 (0.399–1.844)	0.693		
VCT	1412	227	16.1	1.226 (0.699–2.151)	0.478		
Hospitals and medical organizations	1241	157	12.7	0.927 (0.525–1.638)	0.794		
**HIV genotype**							
CRF01_AE	1399	165	11.8	1	Ref	1	Ref
CRF07_BC	1018	170	16.7	1.499 (1.190–1.890)	0.001*	1.365 (1.078–1.730)	0.009^#^
CRF08_BC	177	20	11.3	0.953 (0.582–1.560)	0.847	0.914 (0.534–1.494)	0.731
CRF55_01 B	100	27	27.0	2.766 (1.728–4.428)	<0.001*	3.061 (1.852–4.947)	<0.001^#^
B	127	10	7.9	0.639 (0.329–1.244)	0.187*	0.669 (0.321–1.252)	0.242
CRF67_01 B	171	37	21.6	2.065 (1.386–3.076)	<0.001*	2.083 (1.374–3.101)	<0.001^#^
CRF68_01 B	90	15	16.7	1.496 (0.839–2.665)	0.172*	1.559 (0.839–2.727)	0.137
CRF105_0107	54	18	34.0	3.739 (2.076–6.737)	<0.001*	3.650 (1.966–6.567)	<0.001^#^
Others	141	14	9.9	0.824 (0.464–1.466)	0.511	0.807 (0.433–1.397)	0.469
**DR or not**							
Not resistant	2950	443	15.0	1	Ref	1	Ref
Resistance	327	33	10.1	0.635 (0.437–0.923)	0.017*	0.649 (0.432–0.947)	0.03^#^
**CD4**^**+**^**T cell count**							
≤200	746	60	8.0	1	Ref	1	Ref
201–350	1100	181	16.5	2.252 (1.654–3.065)	<0.001*	2.150 (1.577–2.965)	<0.001^#^
351–500	773	142	18.4	2.573 (1.867–3.545)	<0.001*	2.382 (1.720–3.333)	<0.001^#^
>501	658	93	14.1	1.882 (1.335–2.652)	<0.001*	1.827 (1.283–2.618)	<0.001^#^

Note: Sequences with any missing variable information were excluded from the statistical analysis, and total number was 3277.

1. *: *P* < 0.2, values acceptable for multi-factors logistic analysis.

2. #: *P* < 0.05, statistically significant values.

3DR: drug resistance; STD: sexual transmitted disease; VCT: voluntary counseling and testing.

## 4 Discussion

HIV-1 genetic transmission network is usually used to demonstrate characteristics of a specific colony, like MMSC or heterosexual infections [13,14]. However, to fully and deeply analyze the genetic transmission network, we integrated all sequences of the new diagnoses from our database and identified the key populations in the network. Previous studies on the genetic transmission network mostly prioritized the largest clusters or clusters that recently enlarged to generate more nodes for public health surveillance [15,16]. However, the risk factors considered in our study were more comprehensive. We prioritized the HIV subtype, transmission route, drug resistance, geographical distribution, and age interval. Each factor can identify a specific risk population.

We chose 0.005 gene distance as a threshold in this study to reflect the probable transmission pattern in the last three years [17]. CRF01_AE is the principal circulating strain in Jiangsu province, accounting for 42.3 % [18]. However, the strain did not form the largest cluster; instead, CRF105_0107, CRF55_01 B, and CRF67_01 B formed the most active clusters with attracting nodes from 2017 to 2018. It suggested that the epidemic of new subtypes should be monitored and considered as a key risk factor, regardless of the cluster size or number. CRF105_0107 was previously identified as a new recombination strain emerging among MMSC in Jiangsu [19]. Similarly, CRF55_01 B and CRF67_01 B also emerged from MMSC [20,21]. However, our results indicated that there were also females in these recombinant type clusters. That is means that these females served as the transmission bridge from the MMSC population to other populations. In our study, CRF105_0107, CRF55_01 B, CRF67_01 B, and CRF07_BC were risk variables instead of CRF01_AE, the primary circulating strain in Jiangsu. All these four subtypes are the recombinants of the B subtype [22,23]. Thus, the gene distance was based on the B subtype. However, this raised doubts about whether the gene distance was appropriate for the HIV-1 genetic transmission network study in CRF01_AE or other subtypes except for the B subtype. Thus, further research is needed to explore the gene distance suitable for different subtypes in the genetic transmission network.

The clusters containing multi-transmission routes were considered a risk priority compared to the largest clusters. In most studies focusing on MMSC, a high-risk behavior population, the group had a relatively constant behavior pattern [24,25]. Multi-transmission clusters portrayed complicated sex patterns, implying a higher risk of transmitting the virus to other sexual groups [[26], [27], [28]]. For example, an IDU female had 5 links to other males in the MMSC category, suggesting heterosexual transmission evolving in MMSC relationship. The IDUs may fall under the sexual transmission route if they abuse drugs that stimulate high-frequency sexual behavior without protection. This risk population can only be identified by constructing the HIV-1 genetic transmission network. Compared to MMSC, other sexual populations are not likely to test for HIV-1, and thus have no idea about their HIV status and could be potential viral transmitters. These populations may be the base of the iceberg, with a large number but hard to identify. In our genetic transmission network, the IDU female connected to two males who described themselves infected through heterosexual transmission. It implied that she maybe a sex worker or one of the five men maybe an undisclosed IDU. In any case, an in-depth investigation and intervention on this female would be beneficial in curbing the spread of HIV. If she was a sex worker, the benefits were even more obvious. A review revealed that in low-prevalence epidemics (≤5 % HIV prevalence), the estimated impact of sex-worker interventions included: 6–100 % infections averted; 0.9–6.2 HIV infections averted per 100,000 adults; 11–94 % and 4–47 % relative reduction in prevalence and incidence, respectively [29]. So even though they were not included in the bigger clusters, they could still represent a major risk factor. To sum up, female infections in the MMSC clusters with more than one edge implied that the individuals were probably contributing to HIV-1 transmission and should be precision intervened. However, the transmission effects played by female infections were probably ignored due to not appearing in the results revealed in genetic transmission network.

The UNAIDS established 2025 Global AIDS strategy targets of “95%-95%–95 %" [20], proposed the “U=U″ view for HIV prevention, and emphasized the vital role of ART success [21,30]. In our study, we found a cluster with drug resistant sites from 2017 to 2018, indicating a steady transmission and a threat to the success of ART. The other DR nodes had a pairwise connection, and the nodes with resistant sites did not form bigger clusters; however, they still represent the crucial populations determining the success of ART. Therefore, these individuals should be paid more attention in ART.

The network showed that the interconnections among the cities of south Jiangsu formed large clusters. However, cities of north Jiangsu interacted with themselves through one-to-one connection, which could also evolve into big clusters when connected to cities of south Jiangsu. Therefore, the city with higher infection cases can be selected for risk behavior analysis. These results suggest that conducting transmission studies in these risk cities can identify infection rates or new potential infections.

The HIV-1 prevalence among the individuals over 60 years old is rising yearly, but only a few literatures are reported. This is probably due to the less attention paid to the individuals over 60 years old because of the degeneration of their health and low sexual activity [31]. Interestingly, individuals over 60 years old easily formed networks in our study compared with the other age groups. However, all individuals over 60 years old only formed pairwise or triple clusters, indicating their low mobility in HIV-1 transmission. We observed two female-female pairwise cluster among the individuals over 60 years old in the network, indicating that several non-reported male infections could connect to the clusters and may be form a big HIV-1 genetic transmission network because female infections over 60 years old were almost infected through sexual transmission between spouses. As reported, old adults are less likely to use protective measures such as condoms and have never had an HIV test [32,33]. These factors make them easy to infect HIV-1. Among persons living with HIV, it is estimated that more than half will be aged 50 years or older in the near future. In high prevalence areas, HIV infection has been present in up to 5 % of persons who died older than 60 years [34]. Therefore, any individual over 60 years old in the genetic transmission network should not be ignored. Any cluster connection, even one-to-one, could provide a clue for identifying possible infections and further preventing subsequent transmissions. Accordingly, harmful education and enlarging HIV-1 testing based on the genetic transmission network should be implemented among older adults to ensure timely diagnosis.

CD4 count is a key index to determine the immune level of HIV-infected individuals. And so far, CD4 counts have also been used to assess late presentation. HIV-infected individuals with CD4 counts below 350 cells/μL are considered as late presenters. In our study, most infections had blood taken immediately after diagnosis, and the median CD4 count was 320 cells/μL, meaning an unoptimistic immune profile and late HIV presentation status. Combining with the age concentrated between 20 and 60 years old, the newly diagnosed infections were considered as sexually active population, so the risk of transmission by these infections is higher and it is easier for them to enter the transmission network.

### 4.1 Limitations of the study

This study was an original research of HIV-1 genetic transmission network. All results were only from laboratory sequences and theoretical analysis. The genetic transmission network role in precision intervention on high-risk populations was still deficiency. This precision intervention is carrying out and the effects on stopping the spread of HIV are still waiting to see.

## 5 Conclusion

Our study elaborated on the characteristics of the HIV-1 genetic transmission network in Jiangsu province. Clusters containing drug resistant nodes and individuals over 60 years old should be intervened, whatever the sizes of clusters nor the numbers of nodes in clusters. CRF01_AE may be ignored in the genetic transmission network based on gene distance <0.005 compared with CRF105_0107, CRF55_01 B, and CRF67_01 B. Besides factors above, we still should pay more attention to high-risk population with active sexual behavior in Jiangsu. Furthermore, identification, testing, and treatment interventions for the key population in the transmission network could help achieve the goal of 95%-95%–95 % from the World Health Organization and push to end AIDS epidemic.

## Data availability statement

Data associated with the study has not been deposited into a publicly available repository and data will be made available on request.

## Funding statement

This research did not receive any specific grant from funding agencies in the public, commercial, or not-for-profit sectors.

## Ethics declaration

Informed consent was not required for this study because all personal information were obtained from the National HIV/AIDS Surveillance System.

## Additional information

No additional information is available for this paper.

## CRediT authorship contribution statement

**Ying Zhou:** Writing - review & editing, Writing - original draft, Formal analysis, Data curation, Conceptualization. **Jing Lu:** Writing - original draft, Methodology, Investigation, Formal analysis, Data curation. **Zhi Zhang:** Writing - original draft, Methodology, Investigation. **Qi Sun:** Software, Investigation. **Xiaoqin Xu:** Visualization, Validation, Project administration. **Haiyang Hu:** Writing - review & editing, Writing - original draft, Formal analysis, Conceptualization.

## Declaration of competing interest

The authors declare that they have no known competing financial interests or personal relationships that could have appeared to influence the work reported in this paper.
